# Supplementary material for: Controlled bio-inspired self-organised criticality
Source: PLoS One. 2022 Jan 24;17(1):e0260016. doi: 10.1371/journal.pone.0260016 (PMC8786161; doi:10.1371/journal.pone.0260016)
Supplement: Attachment — Submitted filename: PONE Criticality - Reply to Reviewer.pdf [file pone.0260016.s001.pdf]

Reply to Reviewer – Controlled Bio-inspired Self-Organised Criticality

Please note that only comments from one reviewer was provided to me, and I have indicated below my response. Changes in the modified manuscript are indicated in ***bold****; the entire introduction was rewritten to reflect the changes requested.*

Reviewer #1: Based on a nonlinear model of a bienzymatic cycle Scheper demonstrated numerically that rate control of chaos (RCC) may be a suitable robust mechanism to produce Self-Organised Criticality (SOC). The theoretical results are beneficial to in-depth understanding of biological control mechanisms. The theme of this paper is very significant and interesting. But there are certainly severe problems which must be solved well before publication.

1. The organizational framework of manuscript is very terrible, especially the section of introduction. The author must rewrite the section to let readers understood the keynotes easily.

   I have rewritten and restructured the introduction section (lines 1-170). I have created subsections to clarify each component within the introduction and added a clarification at the end of the introduction to explain the structure better. The aim is to explain the steps that lead to the results, starting with the need for SOC, why it is important to be stable controllable, the applicability of SOC within biosystems, and how this then relates to homeostasis. Other parts have also been revised to clarify the language.

2. The related methods used to generate SOC in biosystems should be introduced in brief to get a bird's-eye view of this area

   I have amended the existing sub-section that relates to this aspect (Criticality in Biosystems, line 22) and added a new sub-section that briefly discusses this. There exist some modern biological engineering attempts to create SOC which is included. Most mechanisms to reliably generate SOC are not biologically relevant (based on stochastic approaches of physics models), or are not deterministic, which is explicitly addressed using my approach to generate SOC.

3. Whether the main results obtained here depends the network structure?

   No, the network has no relevance on these results, as it is just a fully connected system using finite difference connections (without self-connections). Figure 3 shows the importance of nonlinearity in connectivity which, apart from RCC, seems to be the only constraints. If connections are missing, the system will not be able to achieve the same amplitude, but this property may be used to create specific dynamic behaviours. This concept has been used (paper under review) to create a deterministic nonlinear representation space for dynamic biological data for machine categorisation.

4. Can RCC be used in another biological chaos model to generate SOC?

   Yes, RCC has already been used to control both biological chaotic models, as well as other non-biological chaotic models (e.g. lasers), all of which are readily controllable using this method. RCC has been used to control networks of oscillators (Grey-Scott, Belousov-Zhabotinsky, Lorenz, Roessler, Fabrikant, etc.), an example is contained in the 2017 paper cited. It has been used to control a glucose bioreactor, and we have made physical demonstrators of the method of toy models (Duffing, inverted pendulum), wind turbines and even a diesel engine. Further results are in preparation for publication.

5. Whether the main conclusions can be maintained if noise exists in the model

   Yes, the perturbations can be noisy; this can be understood by considering that noise causes fast perturbations to occur during the entire simulation. These perturbations are driving each orbit away from the stabilised controlled orbit, but the control maintains the oscillation in the same neighbourhood. In a network of oscillators, this effect can be smoothed out over all oscillators. This property of Rate Control of Chaos is explored in the cited paper Biologically Inspired Rate Control of Chaos.

6. The author should carefully check the clerical errors and mistakes, such as “Given that he primary”.

   Thank you for spotting these, I have carefully checked the paper, and I hope to have caught them all.

Tjeerd olde Scheper – Oxford 23 September 2021
